# Sepsis information-seeking behaviors via Wikipedia between 2015 and 2018: A mixed methods retrospective observational study

**DOI:** 10.1371/journal.pone.0221596

**Published:** 2019-08-22

**Authors:** Craig S. Jabaley, Robert F. Groff, Theresa J. Barnes, Mark E. Caridi-Scheible, James M. Blum, Vikas N. O’Reilly-Shah

**Affiliations:** 1 Department of Anesthesiology, Emory University, Atlanta, Georgia, United States of America; 2 Anesthesiology Service Line, Atlanta Veterans Affairs Medical Center, Decatur, Georgia, United States of America; 3 Department of Biomedical Informatics, Emory University, Atlanta, Georgia, United States of America; 4 Department of Anesthesiology, University of Washington, Seattle, Washington, United States of America; Clemson University, UNITED STATES

## Abstract

Raising public awareness of sepsis, a potentially life-threatening dysregulated host response to infection, to hasten its recognition has become a major focus of physicians, investigators, and both non-governmental and governmental agencies. While the internet is a common means by which to seek out healthcare information, little is understood about patterns and drivers of these behaviors. We sought to examine traffic to Wikipedia, a popular and publicly available online encyclopedia, to better understand how, when, and why users access information about sepsis. Utilizing pageview traffic data for all available language localizations of the sepsis and septic shock pages between July 1, 2015 and June 30, 2018, significantly outlying daily pageview totals were identified using a seasonal hybrid extreme studentized deviate approach. Consecutive outlying days were aggregated, and a qualitative analysis was undertaken of print and online news media coverage to identify potential correlates. Traffic patterns were further characterized using paired referrer to resource (i.e. clickstream) data, which were available for a temporal subset of the pageviews. Of the 20,557,055 pageviews across 65 linguistic localizations, 47 of the 1,096 total daily pageview counts were identified as upward outliers. After aggregating sequential outlying days, 25 epochs were examined. Qualitative analysis identified at least one major news media correlate for each, which were typically related to high-profile deaths from sepsis and, less commonly, awareness promotion efforts. Clickstream analysis suggests that most sepsis and septic shock Wikipedia pageviews originate from external referrals, namely search engines. Owing to its granular and publicly available traffic data, Wikipedia holds promise as a means by which to better understand global drivers of online sepsis information seeking. Further characterization of user engagement with this information may help to elucidate means by which to optimize the visibility, content, and delivery of awareness promotion efforts.

## Introduction

Sepsis has been defined as a potentially life-threatening dysregulated host response to infection and imparts a significant worldwide burden of disease [1,2]. Early recognition and intervention have emerged as cornerstones of successful management, and impactful efforts have been undertaken to disseminate best practice information to the healthcare community [3–5]. As the impact of sepsis in resource-poor areas is likely under-appreciated, it has therefore been named a global health priority by the World Health Organization [6,7]. In conjunction with these efforts there have emerged campaigns by governmental and non-governmental organizations to raise public awareness of sepsis both to hasten its recognition in the community, and other pre-hospital settings, and highlight its importance with regard to research funding, regulation, charitable giving, and public-private partnerships [8–11]. Assessing the net impact of these information dissemination efforts represents a significant challenge. Survey findings have been mixed and, more broadly, may be limited in their scope, timeliness, and granular insight into drivers of awareness or information seeking [12–17].

Utilization of online healthcare information is increasingly common and represents one means by which to gain insight into information-seeking behaviors [18,19]. Interest has increasingly grown in the utilization of “big data” from these relatively novel sources to conduct a wide range of investigations, including the potential prediction of disease outbreaks [20,21]. Seasonal trends in web traffic for asthma and influenza, for example, have been found to generally mimic natural variances in disease incidence and severity [22–24]. Specific to sepsis, both public awareness campaigns and media coverage of high-profile events, such as celebrity deaths, have been previously associated with heightened search engine utilization [25]. Additionally, families of critically ill patients have been reported to utilize online healthcare information to learn more about disease processes, including sepsis [26,27]. However, access to web traffic information is almost always unavailable to researchers or other members of the public owing to concerns surrounding protection of intellectual property, business use cases, and user privacy [28]. Google (Alphabet Inc., Mountain View, CA, USA), for example, makes available to the public only relative search volumes and reduces their temporal resolution in response to searches spanning more than a few months. Additionally, geographic and socioeconomic search engine preferences limit the generalizability of findings from any given set of utilization data.

These considerations similarly impact researchers seeking to examine traffic patterns to and from user end-destinations, such as informational web pages. Wikipedia (Wikimedia Foundation Inc., San Francisco, CA, USA) is a publicly curated online encyclopedia that, in its English localization alone, currently consists of over 47 million pages and sees, on average, over 250 million pageviews daily [29]. Wikipedia has been previously identified as an influential source of online science and technology information with both cyclic and ad-hoc utilization around periods of heightened media attention to relevant topics [30]. The Wikimedia Foundation balances its goals of transparency and protection of user data by providing aggregate, anonymized utilization data through a variety of public tools [31]. Given the global popularity of Wikipedia and its publicly available granular utilization data, examination of its traffic patterns offers a unique opportunity to examine the temporal association between media coverage of diseases, awareness campaigns, and other events with online information-seeking behaviors on a large scale.

We hypothesized that examination of sepsis-related Wikipedia traffic may be able to highlight the types of events that prompt the public to learn more about the condition. This has potentially wide-ranging impact for researchers, clinicians, and agencies that may wish to either promote disease awareness during periods of heightened public attention or critically examine the impact of their efforts in a more dynamic and wide-ranging fashion than that offered by traditional methodologies, such as surveys. We sought to examine user traffic for the sepsis and septic shock Wikipedia pages, referred to as pageviews, identify periods of heightened traffic, and further characterize relevant user traffic patterns. Based on previous work, we further hypothesized that media coverage of high-profile events would be correlated with heightened online information seeking; in particular, we wished to test the null hypothesis that officially sponsored public awareness campaigns (e.g. Sepsis Awareness Month) would not impact traffic to Wikipedia.

## Methods

A written exemption from review was granted by the Emory University Institutional Review Board on the basis that the present work does not constitute research with human subjects. Reporting follows applicable elements of the Strengthening the Reporting of Observational Studies in Epidemiology (STROBE) statement guidelines [32].

### Sepsis and septic shock pageview analyses

Wikipedia can be conceptualized as a group of highly inter-related, language-specific online encyclopedia projects designed to curate and deliver content [33]. These efforts are coordinated through the broader WikiMedia Foundation, and at the time of writing there are 294 active, language-specific Wikipedia localizations [34,35]. Aggregate Wikipedia traffic and utilization data are made available through a suite of publicly accessible tools developed and hosted by the Wikimedia Foundation [31]. Each primary entry in Wikipedia is referred to as a page, and we first examined user traffic to the Wikipedia pages for sepsis and septic shock. Sepsis and septic shock pageview counts by users on all platforms (i.e. desktop and mobile), across available languages between July 1, 2015 and June 30, 2018 were extracted using the Langviews tool, which provides daily pageview counts. Data were aggregated down to a single univariate time series of total daily pageviews for both sepsis and septic shock for subsequent quantitative analysis. Page titles are largely standardized across localizations in different languages to promote standardization and facilitate interoperability. As such, the Langviews tool, for any given Wikipedia page, also reports pageviews on a per-language basis.

#### Statistical methods and time series analyses

Preliminary examination of this univariate pageviews time series demonstrated modest seasonality and a slight trend component. We opted for a seasonal hybrid extreme studentized deviate approach (S-H-ESD) for outlier detection, which was designed for the analysis of web traffic data of varying temporal resolution [36,37]. We have previously employed S-H-ESD for the analysis of daily Wikipedia pageview traffic [22]. S-H-ESD uses a modified Loess approach to additive seasonal decomposition with piecewise medians replacing the conventional trend component. By utilizing medians and the mean absolute deviation as measures of central tendency, versus the mean and standard deviation in a conventional ESD procedure, this approach is robust against large outliers [38]. Given the marginal trend component, a median span duration of 182 days was selected. After fitting, residuals did not demonstrate heteroscedasticity, or unequal variance (i.e., scatter), either when visually examined or by the Breusch-Pagan test, which tests the null hypothesis that variance amongst the residuals is equal. Therefore, data transformations were not employed. ESD iteratively examines the residuals for outlying values against a normal distribution, and we selected a conservative alpha of 0.01 given (a) use of noisy daily data and (b) the desire to identify and characterize only meaningful outliers. The practical end-result of this approach is the identification of daily pageview counts that are significantly outlying, accounting for seasonal trends and variable central tendency as measured by 182 day median spans.

Time series analyses were conducted with the AnomalyDetection (version 1.0) and anomalize (version 0.1.1) packages in R (version 3.5.3) via RStudio (version 1.1.463) [39,40]. Figures were generated to (a) highlight the distribution of pageviews for sepsis and septic shock, in aggregate, by language, and (b) graphically depict the univariate pageviews time series and highlight outlying daily values. Figures were generated using the ggplot2 (version 3.1.1) and ggrepel (version 0.8.0) packages for R [41,42].

### Qualitative examination of outlying pageview epochs

Sequential days with outlying pageview values were aggregated and treated as related temporal periods, or epochs, based on prior work demonstrating lagged relationships [22,25]. As outlying epochs were hypothesized to correlate with news media coverage of sepsis, we searched for relevant media coverage during these epochs from two sources. For online coverage of sepsis, Google News was queried for the date of the outlying epoch; the day before and the day after the epoch were also included to account for the impact of varying time zones and lagged effects. For print media coverage of sepsis, Factiva was similarly queried (Factiva, LLC; Dow Jones & Company, New York, NY USA). Google News was chosen due to its comprehensive indexing of online news coverage, public accessibility in support of reproducibility and extensibility of our methodology, and ability to search historically with daily granularity. Factiva, although not publicly accessible, indexes print media from over 30,000 sources across 28 languages and is therefore one of the most comprehensive print content aggregators. We have previously utilized these two sources in tandem for related investigations [22]. As English localization pageviews accounted for the greatest proportion of represented languages, searches were first conducted in English. When media correlates could not be readily identified, per-language subsets of the data were examined to modify the search strategy using terms previously identified to search for sepsis-related information in different languages [25]. Where applicable, media coverage of scientific investigations was attributed through examination of the full text for citations.

### Further characterization of user behavior

To facilitate a comparison to other prominent medical conditions, the Langviews tool was similarly utilized to extract pageview counts for myocardial infarction and influenza by users on all platforms across all localizations between July 1, 2015 and June 30, 2018.

The software platform underlying Wikipedia allows for the creation of categories, which are meant to aid in aggregation of related pages and media to support the longitudinal generation of a hierarchical knowledge structure [43]. To ascertain the extent to which searches for sepsis and septic shock compared to those for other critical care topics, the Wikimedia Massviews tool was utilized to extract data for pages within the category of critical care for the English language localization [44].

Beginning in January of 2015, The Wikimedia Foundation began to release data intended to facilitate conceptualization of user browsing behavior [45,46]. These monthly data sets consisted of paired counts between referrers and resources. For example, the number of users following a link from one Wikipedia page to another. When examined in aggregate, it is therefore possible to ascertain user traffic patterns both leading to a given page and from a given page, commonly referred to as a clickstream. These large datasets (representing over 25 million pairs from about 6.8 billion requests, monthly, as of 2016) were released sporadically and in variable formats until November 2017, at which point the format was standardized and the releases routinized. Additional technical details about the manner in which Wikipedia generates clickstream data are available online [47,48]. Of note, some referrer data is unavailable due to the methodology underlying its generation, including refererless traffic from clients utilizing a HTTPS protocol. We aggregated and analyzed the 8 consecutively available monthly datasets representing English localization traffic pairs from 11/1/2017 to 6/30/2018. The datasets were parsed using SQL in R owing to their large size with the read.csv.sql() function of the sqldf package(version 0.4–11) [49]. As visualization was confounded by a large number of unique, but interrelated, inbound linkages from within Wikipedia, we manually reviewed and then aggregated the represented Wikipedia pages into broad categories via independent review by two authors (CSJ and RG) with discrepant opinions refereed by a third (VORS). For example, Wikipedia pages about gangrene and bacteremia were categorized as conditions. Thereafter a Sankey diagram was generated using JavaScript code derived from Google Charts [50].

## Results

**Fig 1:**
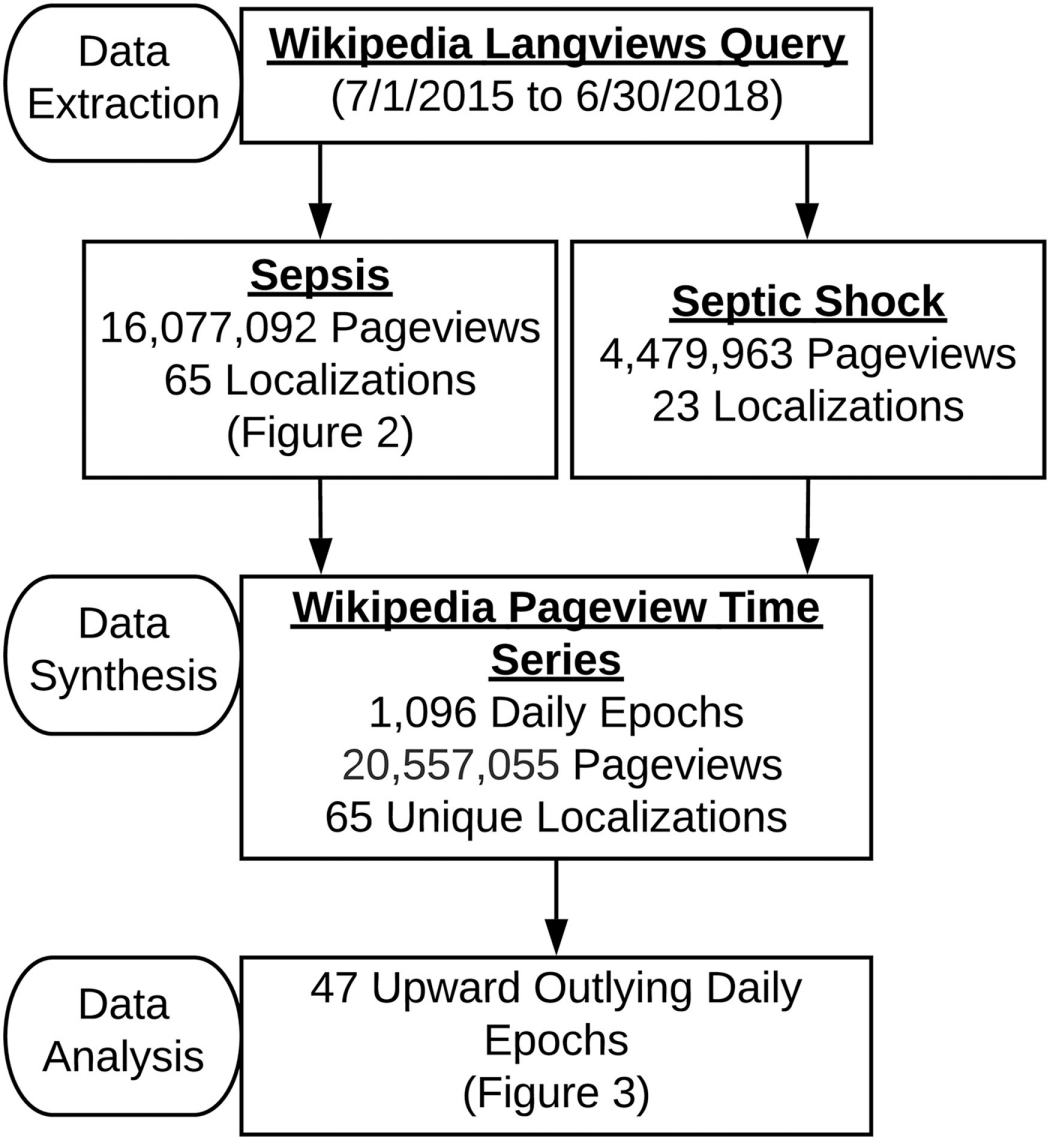
Sepsis and septic shock Wikipedia pageview data workflow. The Wikimedia Langviews tool provides aggregate traffic data for a given Wikipedia page across all language localizations.

**Fig 2:**
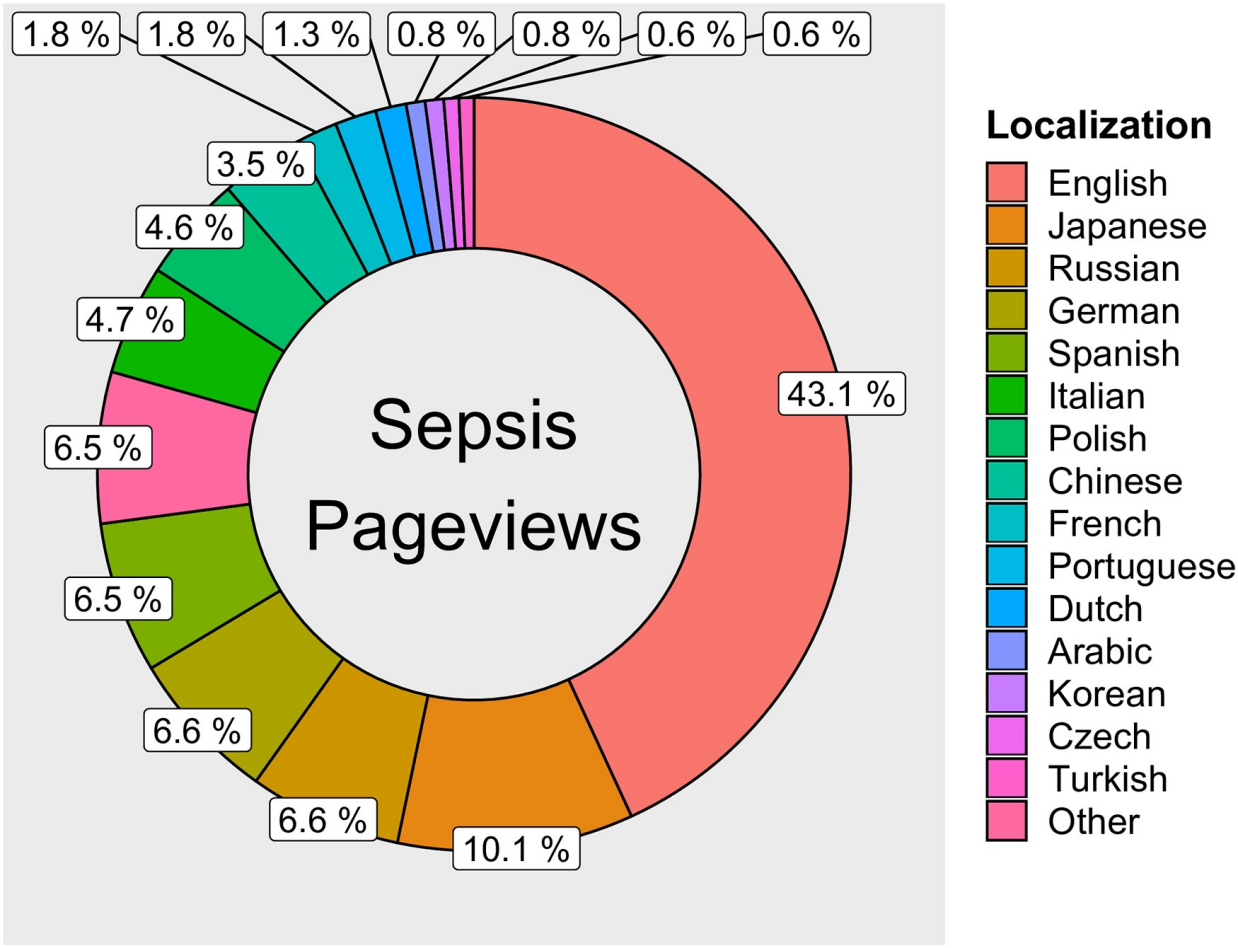
Sepsis Wikipedia user pageviews by language localization. To improve legibility, only the top 15 languages are depicted. The remaining 50 are summarized in the “Other” category. The legend appears in descending numerical order with “Other” listed last.

**Fig 3:**
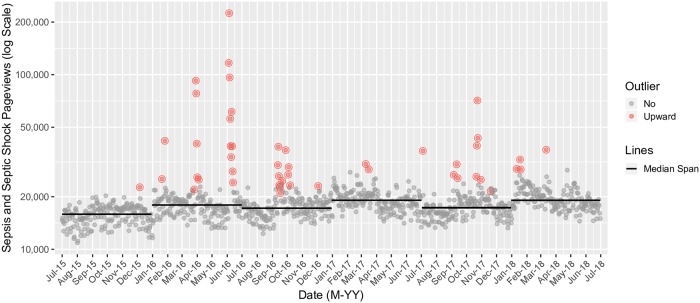
Sepsis and septic shock user pageview traffic (2015 to 2018). Data extraction spanned July 1, 2015 to June 30, 2018. Outliers with observed values significantly (alpha = 0.01) exceeding those expected are highlighted. Outliers with observed values significantly below those expected were not sought and are not depicted. Median spans are displayed as horizontal black lines (see Methods). The Y axis employs a log scale to improve legibility in the setting of large outliers within the time series. Suggested correlates for outlying values are presented in Table 1. Pageviews across all available languages are depicted in the figure.

During the dates of interest, there were 20,557,055 views of the sepsis and septic shock Wikipedia pages across 65 languages (Fig 1, S1 and S2 Files), with an average of 18,756 views per day. In comparison to other prominent diseases, myocardial infarction had 14,631,135 views across 98 languages, and influenza had 14,470,222 views across 76 languages. The sepsis page was available in more languages than the septic shock page (N = 65 versus 23), with pageviews in English being most common (43.1%); followed by Japanese (10.1%); and then Russian, German, Spanish, and Italian accounting for more than 5% each (Fig 2). Out of the examined 1,096 daily pageview values, 47 were found to significantly exceed those expected (Fig 3).

Online and print news media correlates were readily identified for all outlying epochs (Table 1). The identified correlates largely centered on celebrities and other persons with sepsis, including high-profile deaths. Contemporaneous awareness promotion efforts around these events were discernible; however, a smaller number of isolated awareness promotion correlates were also identified. While variable in year, these primarily occurred in the month of September, which is Sepsis Awareness Month. Media coverage of two peer-reviewed publications was identified in addition to one policy statement and one Morbidity and Mortality Weekly Report from the United States Centers for Disease Control (CDC) [51–54].

**Table 1:** Outlying Wikipedia sepsis and septic shock epochs with potential media correlates (2015 to 2018).

Outlying Epoch	Date(s) (M/D/YYYY)	Highest Observed Pageview	Rank (of 25)	Potential Correlates
1	12/6/2015	22,657	23	UK National Health Service (NHS) apology for pediatric sepsis death (Ben Condon)
2	1/20/2016	25,243	17	Coverage of host gene expression classifiers [51] Adult sepsis case (Raimonds Vējonis)
3	1/26/2016	41,784	4	NHS apology for pediatric sepsis death (William Mead)
4	3/26/2016	22,000	24	Sepsis survivor coverage (Andressa Urach and Matthew Parkes)
5	3/29/2016–4/1/2016	92,305	2	Death of Patty Duke from abdominal sepsis
6	4/4/2016	25,048	18	Ongoing coverage of Patty Duke
7	6/4/2016–6/13/2016	224,927	1	Death of Muhammad Ali from sepsis
8	9/12/2016–9/17/2016	38,644	5	Inaugural World Sepsis Congress Fifth World Sepsis Day US Centers for Disease Control (CDC) awareness efforts
9	9/21/2016	24,674	20	UN draft declaration on antimicrobial resistance [52] Meningitis Awareness Week campaign in the UK
10	9/28/2016	36,984	7	Sepsis promotion efforts in the UK (Melissa and William Mead) Pediatric sepsis death in Russia
11	10/3/2016–10/4/2016	29,553	12	Adult sepsis deaths (Lucinda Smith and Jayaram Jayalalithaa)
12	10/7/2016	23,256	21	Coverage of a US Pediatric sepsis death linked to nutritional deficiency
13	12/3/2016	23,023	22	Coverage of childhood neglect and attempted murder (Tiffany Alberts) Death of a veteran in a VA facility from sepsis (Owen Reese Peterson)
14	3/10/2017	30,768	10	National Institute for Health and Care Excellence (NICE) sepsis 1-hour intervention mandate in the UK
15	3/16/2017	28,667	14	Sepsis survivor coverage (Kevin Breen)
16	7/4/2017	36,600	8	Coverage of CDC MMWR concerning dehydrated placenta [53] Adult sepsis death (Shinji Mori) Pediatric sepsis case in Taiwan
17	9/5/2017	26,685	15	Ireland Health Service Executive (HSE) national sepsis report Sepsis awareness month coverage
18	9/11/2017	30,664	11	NHS trusts sepsis performance Ongoing sepsis awareness coverage
19	9/13/2017	25,532	16	Sixth World Sepsis Day
20	10/21/2017–10/24/2017	71,140	3	Adult sepsis deaths (Rodolfo Torres Rojas, Rasiklal M Dhariwal, death in Korea linked to musician Choi Si-won)
21	10/30/2017	25,030	19	Sepsis in the aftermath of Hurricane Maria Coverage of geographic outcome disparities in the UK
22	11/19/2017	21,555	25	Adult sepsis death (José Manuel Maza)
23	1/11/2018	28,857	13	Young adult sepsis death (Kyler Baughman) following influenza
24	1/18/2018–1/19/2018	32,630	9	Adjunctive steroids in septic shock [54] Adult sepsis deaths (Alyssa Alcaraz and Olivia Nova)
25	3/12/2018	37,142	6	Adult sepsis misdiagnosis (Magdalena Malec) Adult sepsis deaths (Oleg Tabakov and Kenneth Flach)

Data extraction spanned July 1, 2015 to June 30, 2018. The highest pageview count per aggregated epoch is depicted and its rank displayed.

**Fig 4:**
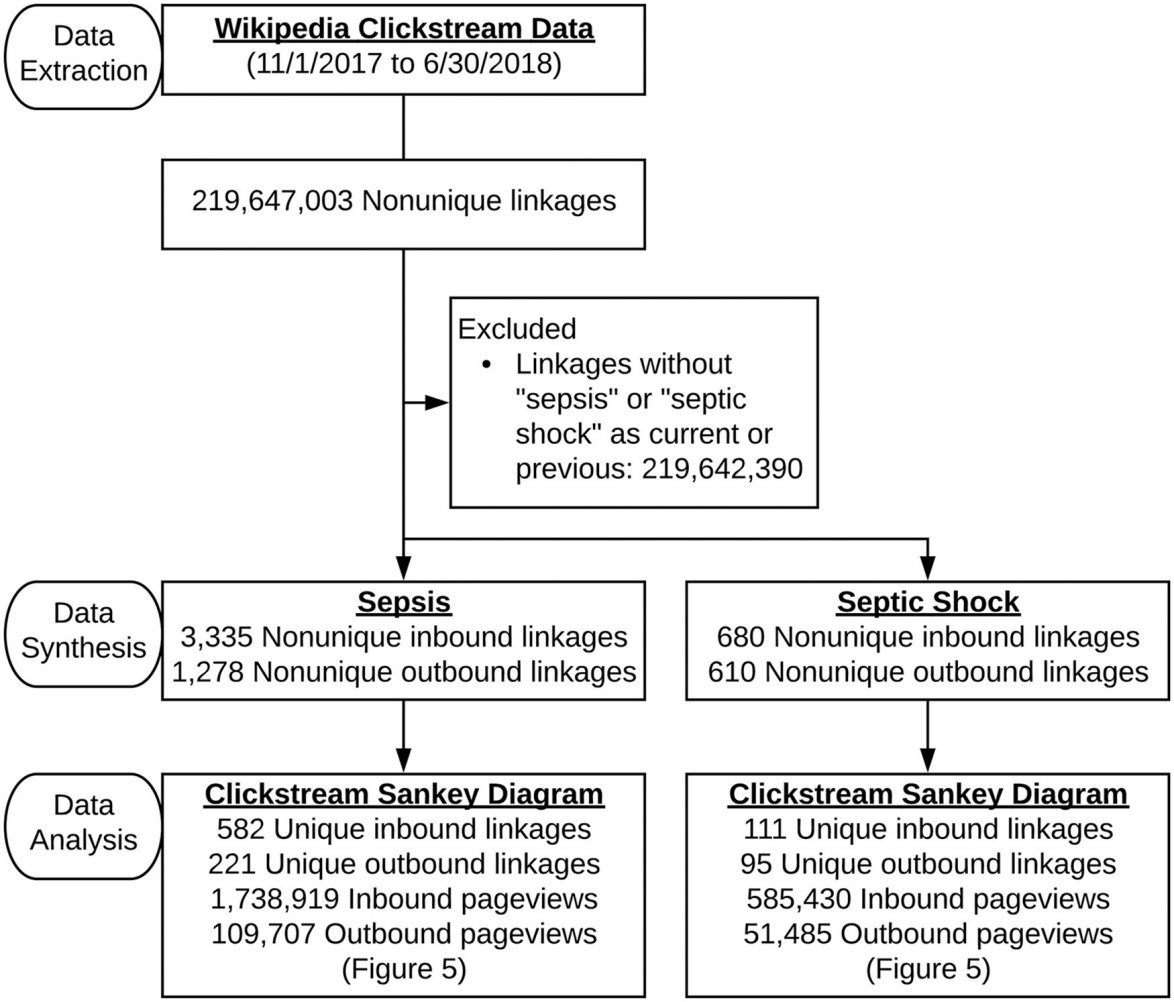
Wikipedia clickstream data workflow. Since November 2017, the Wikimedia Foundation has released monthly aggregate user browsing behavior in the form of consistent, large inbound and outbound linkage datasets representing English language pageview traffic.

**Fig 5:**
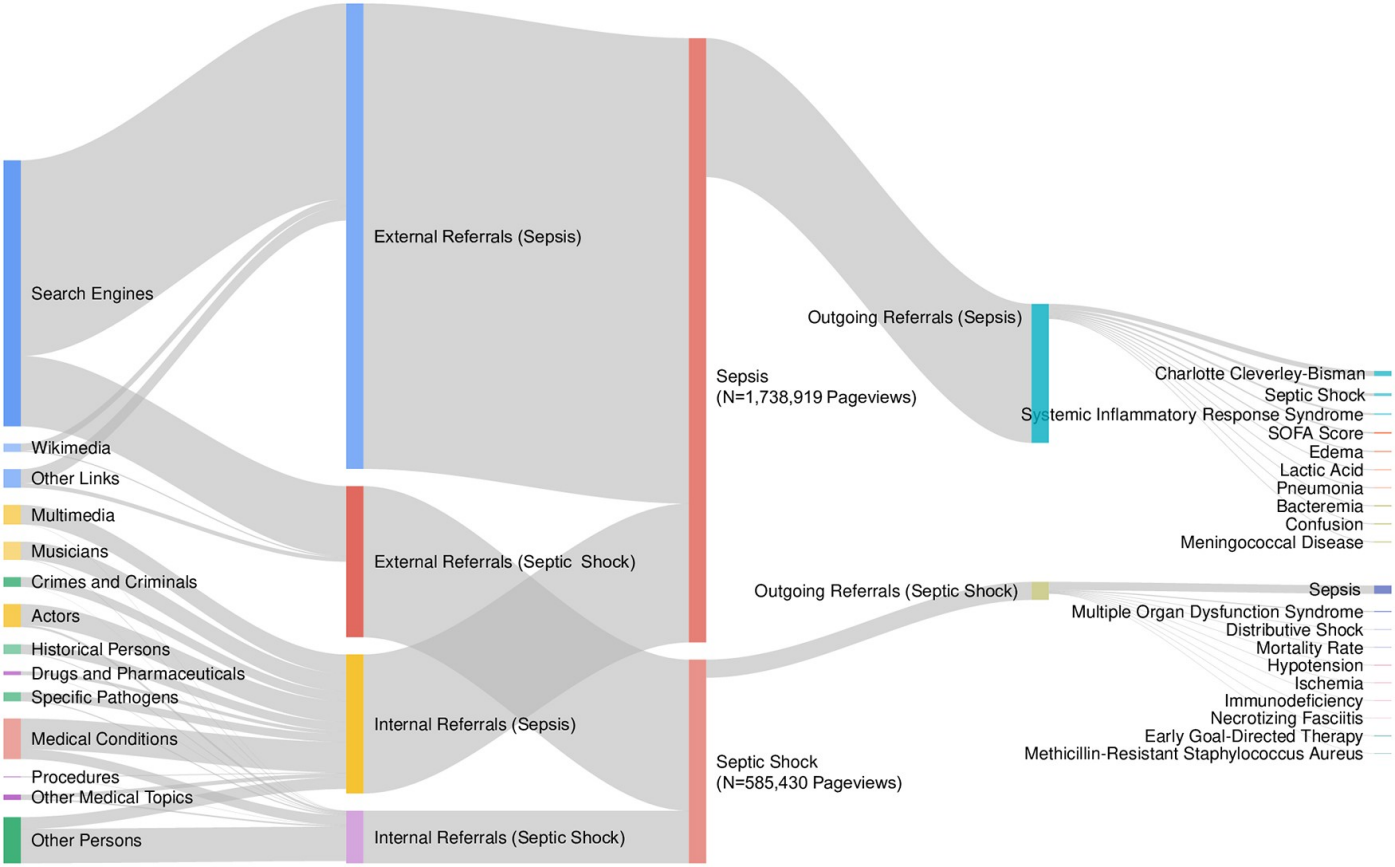
Sepsis and septic shock Wikipedia clickstream sankey diagram (November 2017 to June 2018). Owing to a large number of unique inbound linkages within Wikipedia, individual Wikipedia pages were aggregated into common categories (see Methods). Only the top 10 outbound Wikipedia pages are listed to improve legibility. Missing data are due to limitations in the underlying data, including refererless traffic from clients utilizing a HTTPS protocol. Source data are available in supporting files.

English localization pageviews of sepsis and septic shock accounted for 18.1% of all pageviews related to critical care as organized by topics (N = 9,646,193/53,155,568; S3 File). Monthly clickstream datasets representing English language pageviews (as stated, limited to the last 8 months of the dates of interest) were parsed and contained a large number of unique inbound linkages (Fig 4, S4–S7 Files). These were manually examined and categorized, with Wikipedia pages pointing to either the sepsis or septic shock pages being largely related to people (e.g. historical persons or celebrities) and medical topics (S8 and S9 Files). These linkages were used to plot a Sankey diagram of Wikipedia traffic for sepsis and septic shock (Fig 5). Search engines accounted for the majority of known external referrals, and the top 10 outgoing referrals largely related to medical topics.

## Discussion

Epochs of outlying sepsis and septic shock Wikipedia pageviews were correlated with relevant online and print news media coverage, which often featured celebrities and other high-profile individuals. Correlates related to sepsis awareness campaigns were identified, but these were less frequent in number and associated with lower total pageview counts. In examining the temporal subset of pageviews where corresponding user traffic pattern data were available, the majority of users accessed sepsis information on Wikipedia via search engines. In conjunction with a low number of recorded internal outbound linkages, which would represent users clicking through to additional Wikipedia topics, these Clickstream findings may suggest that the preponderance of sepsis and septic shock pageview traffic was the result of specific information-seeking by users rather than the result of casual Wikipedia browsing. This finding should be interpreted with caution owing to the monthly granularity of the Clickstream data, which precludes analysis around specific events that may drive information-seeking. Although comparator data is lacking, Wikipedia looks to be a popular venue for sepsis information seeking with over 20 million user pageviews during the three years examined. In contrast, sepsis did not feature among the top 20 pages accessed on CDC.gov in 2017, which would place its total pageviews as less than 1.76 million [55]. Public tax filings from the Sepsis Alliance note that it received “more than 1 million visits” in 2016, again suggesting that Wikipedia may be a major end-point of sepsis information seeking [56]. These findings may speak to several factors in isolation or in combination: Wikipedia’s overall popularity, user trust in its content, and its global presence [57–59]. Pageviews for sepsis were more common than those for septic shock, which could be due to the former’s greater availability across more languages and possibly because familiarity with the latter is largely confined to the medical community [12].

Though similar patterns in Google search trends were previously identified, important differences merit comment [25]. Search volumes for influenza and myocardial infarction were previously shown to exceed those related to sepsis; however, sepsis appears to be more frequently viewed than these conditions on Wikipedia. Examination of clickstream data for influenza and myocardial infarction may help to better elucidate the user behaviors driving these disparate information-seeking patterns. In contrast to Wikipedia pageviews for asthma, those for sepsis and septic shock demonstrated less seasonal variability, which may be a product either of differences in the underlying disease states themselves or non-episodic baseline user behaviors [22]. Although the identified media correlates should be viewed as speculative, extremely high-profile events–such as the sepsis-related deaths of Muhammad Ali in June 2016 and Patty Duke in late March 2016 –corresponded to significant episodic information seeking. The impact of peer-reviewed publications and other scientific communication did not emerge as clearly, which likely reflects the extent to which the public versus the medical community engage with Wikipedia to find sepsis-related information.

Given its importance from a global health standpoint, a better understanding of when, why, and how the public and healthcare providers alike seek out information about sepsis should be a core priority underlying awareness promotion efforts. Such information can help to better inform the content and timing of efforts and to gauge their impact. The present investigation suggests that Wikipedia is utilized globally to learn about sepsis. Although beyond the scope of the present work, content curation by organizations working to raise awareness may therefore be of some importance to ensure that the public has access to timely and high-quality information. In addition, on the basis of presented information-seeking patterns, these same organizations may be well advised to augment their outreach efforts in concert with high profile cases of sepsis in the popular media.

Our investigation has several strengths. Compared to other publicly available web traffic data (i.e. Google Trends), Wikipedia pageviews are reported in absolute values on a daily basis therefore offering both improved granularity and comparability. As search engine preferences are likely impacted by a number of factors, examination of Wikipedia and other popular destinations with curated content may provide more clarity as to information both sought and delivered. Similar methodology applied to other disease states of interest may yield valuable insights; notably, our investigation of information seeking related to asthma revealed that major public awareness campaigns did not seem to impact Google search traffic.

Our investigation has several weaknesses. We examined only pageviews for sepsis and septic shock, and users may have sought out related information on Wikipedia that was not captured by our analysis. This approach is supported by our findings that certain sepsis-related events are associated with information-seeking specific to sepsis, the relative prominence of sepsis and septic shock pageviews amongst all Wikipedia pages related to critical care, and the clickstream analysis suggesting a low incidence of casual browsing to the sepsis and septic shock pages. The reported media coverage correlates were identified through a qualitative methodology, and causal inference cannot be established. Reported correlates may not be comprehensive owing to challenges inherent to their retrospective identification. In particular, tools meant to search historical print media are limited in their accessibility, scope, and international coverage. As such, we did not undertake a comparative analysis of the impact of online versus print media coverage of events as neither may be complete based on our methodology. While the majority of identified correlates concerned events in the US, UK, or Ireland, the present approach identified more international events compared to our prior investigations; it is unclear if this represents bias due to our methodology or represents a more global reflection of drivers of information seeking. Additionally, our qualitative approach focused primarily on identification of correlates in the English language followed by those in other languages, and subsequent investigations could be strengthened or further localized through detailed examination of the full pageviews data set (S1 and S2 Files). User pageview traffic pattern data were available only for a subset of the dates of interest, and its monthly granularity precluded specific examination of changes at the level of an outlying epoch. However, these data do offer important insights into overall user behavior that have not been previously elucidated. As with any user traffic data, there are potential limitations inherent to its processing, aggregation, and reporting. In this instance, these limitations are mitigated by transparent reporting and meticulous documentation on the part of the data provider [60].

## Conclusions

Online and print news media coverage of events related to sepsis appear to heighten their visibility, prompting online information seeking via Wikipedia. The present work represents an initial effort towards refining and developing methodologies to assess such behaviors. Further work in this area will help to strengthen the case for contemporaneous awareness promotion efforts as well as focus content delivery and curation on the part of interested organizations.

## Supporting information

S1 File Wikipedia Langviews sepsis data. Dates spanning July 1, 2015 to June 30, 2018. Data derived from Wikipedia traffic and provided via the Wikimedia Foundation Langviews tool. https://tools.wmflabs.org/langviews/.
(XLSX)

S2 File Wikipedia Langviews septic shock data. Data spanning July 1, 2015 and June 30, 2018. Data derived from Wikipedia traffic and provided via the Wikimedia Foundation Langviews tool. https://tools.wmflabs.org/langviews/.
(XLSX)

S3 File Wikipedia Massviews data. Data spanning July 1, 2015 and June 30, 2018 for the category of critical care. Data derived from Wikipedia traffic and provided via the Wikimedia Foundation Massviews tool. https://tools.wmflabs.org/massviews.
(CSV)

S4 File Wikipedia clickstream data sepsis inbound. Data spanning November 1, 2017 and June 30, 2018. Data derived from Wikipedia traffic and provided via the Wikimedia Foundation Analytics Team. https://meta.wikimedia.org/wiki/Research:Wikipedia_clickstream#Releases.
(CSV)

S5 File Wikipedia clickstream data sepsis outbound. Data spanning November 1, 2017 and June 30, 2018. Data derived from Wikipedia traffic and provided via the Wikimedia Foundation Analytics Team. https://meta.wikimedia.org/wiki/Research:Wikipedia_clickstream#Releases.
(CSV)

S6 File Wikipedia clickstream data septic shock inbound. Data spanning November 1, 2017 and June 30, 2018. Data derived from Wikipedia traffic and provided via the Wikimedia Foundation Analytics Team. https://meta.wikimedia.org/wiki/Research:Wikipedia_clickstream#Releases.
(CSV)

S7 File Wikipedia clickstream data septic shock outbound. Data spanning November 1, 2017 and June 30, 2018. Data derived from Wikipedia traffic and provided via the Wikimedia Foundation Analytics Team. https://meta.wikimedia.org/wiki/Research:Wikipedia_clickstream#Releases.
(CSV)

S8 File Wikipedia clickstream data sepsis inbound manual categorization. See manuscript methods.
(CSV)

S9 File Wikipedia clickstream data septic shock inbound manual categorization. See manuscript methods.
(CSV)
